# A contemporary research topic: manipulative approaches to human brain dynamics

**DOI:** 10.3389/fnhum.2015.00118

**Published:** 2015-03-06

**Authors:** Keiichi Kitajo, Takashi Hanakawa, Risto J. Ilmoniemi, Carlo Miniussi

**Affiliations:** ^1^Rhythm-based Brain Information Processing Unit, RIKEN BSI-TOYOTA Collaboration Center, RIKEN Brain Science InstituteWako, Japan; ^2^Laboratory for Advanced Brain Signal Processing, RIKEN Brain Science InstituteWako, Japan; ^3^Department of Advanced Neuroimaging, Integrative Brain Imaging Center, National Center of Neurology and PsychiatryKodaira, Japan; ^4^Department of Neuroscience and Biomedical Engineering, Aalto University School of ScienceEspoo, Finland; ^5^Cognitive Neuroscience Section, IRCCS Centro San Giovanni di Dio FatebenefratelliBrescia, Italy; ^6^Neuroscience Section, Department of Clinical and Experimental Sciences, University of BresciaBrescia, Italy

**Keywords:** transcranial magnetic stimulation, transcranial electrical stimulation, electroencephalography, TMS-EEG, multimodal imaging

A long-standing issue in neuroscience is how we can evaluate the internal states of the intact human brain and its dynamics. Indeed, significant progress has been made by combining different methods in the so-called multimodal imaging approach, providing an empirical way to directly and effectively measure the brain state and its complex responses via manipulative approaches (Ilmoniemi et al., 1997; Noguchi et al., 2003; Mochizuki et al., 2006; Siebner et al., 2009). The results obtained from integrating different methods offer new, interesting scenarios and are having a revitalizing impact on experimental and clinical neuroscience, as the obtained results are more than the sum of the results provided by the single techniques when used in isolation.

In this Research Topic, “Manipulative approaches to human brain dynamics,” we aim to highlight how these newly emerging techniques for non-invasive stimulation of the human brain (NIBS), combined with concurrent recordings of neural activity, contribute to the understanding of brain functions and neural dynamics. We mainly focus on transcranial magnetic stimulation (TMS), transcranial direct current stimulation (tDCS), transcranial alternating current stimulation (tACS), and transcranial random noise stimulation (tRNS), especially in combination with simultaneous recordings of human brain activity such as electroencephalography (EEG), functional magnetic resonance imaging (fMRI), and near-infrared spectroscopy (NIRS). We also consider theoretical, methodological, and modeling works to understand how these manipulative methods function.

Among the included papers, a number of them employed TMS-EEG co-registration methods and analyzed TMS-evoked potentials (TEP). Veniero et al. (2013) demonstrated that short-latency TEPs (P5–N8) induced by stimulation of the primary motor cortex (M1) were modulated after conditioning of the premotor cortex by repetitive TMS (rTMS). Their results suggest that the short-latency TEPs have a cortical origin, and can be used for evaluating the direct reactivity of the cortex. Yamanaka et al. (2013) compared two long-latency components of TEPs elicited by M1 stimulation, N100, and a later positive component (LPC), at preparatory, executive, and inhibitory stages of a go/stop (or “go/no go”) task. They observed different modulation of N100 between go and stop trials, but LPC did not show such differential modulation. These results suggest that TMS-induced neuronal responses in M1 and subsequent propagation of neural reactions to other cortical areas observed as TEPs might show functional changes according to task demand. Zanon et al. (2013) investigated the propagation of TEPs when stimulating the left dorsal premotor cortex, and found prominent propagation mainly to the contralateral sensorimotor and frontal cortices at about 130 ms after TMS. They also found propagation to the posterior visual regions between 70 and 130 ms after TMS. This study revealed connectivity between the left dorsal premotor cortex and other fronto-parietal regions. Akaishi et al. (2013) tested task-related modulation of effective connectivity during perceptual decision making. They demonstrated that short-latency (20–40 ms) TEPs elicited by TMS to the frontal eye field were modulated as a function of the time to behavioral responses, whereas TEPs elicited by TMS to the ventral prefrontal cortex changed depending on whether the response was correct or not. These studies show that TEPs are useful for probing the reactivity of a cerebral cortical area and also the connectivity between cortical areas, which can be modulated according to task demand.

Some TMS-EEG papers have provided new and interesting perspectives on how TMS can modulate human brain activity in relation to neural dynamics and information flow. Mutanen et al. (2013) beautifully demonstrated that single-pulse TMS evoked changes in the brain-state dynamics. Innovative quantitative measures in their work clearly showed that TMS-induced brain-state dynamics differed from the spontaneous dynamics present before TMS. Kawasaki et al. (2014) provided evidence of TMS-induced modulation of oscillatory brain dynamics and directed information flow. They demonstrated that single-pulse TMS induced the global propagation of transient phase resetting and enhanced information flow from the TMS-targeted visual area to the motor area. These papers indicate how TMS can manipulate neural dynamics and information flow in the intact human brain.

Two sleep-related works have investigated the modulation of oscillatory activity with TMS. Manganotti et al. (2013) investigated single-pulse TMS-induced modulation of ongoing neural oscillations estimated by EEG wavelet power analyses during wakefulness, sleep deprivation, and sleep. They found a reciprocal effect on slow and fast oscillations in response to TMS after sleep deprivation and sleep. Pellicciari et al. (2013) tried to understand the neurophysiological mechanisms of rTMS treatment for depression. They showed that 2 weeks of bilateral rTMS over the dorsolateral prefrontal cortex of depressed patients induced a decrease in alpha activity over the left prefrontal cortex during REM sleep, and this neurophysiological change was significantly associated with the final clinical outcome.

One paper reported fMRI in combination with TMS. Shitara et al. (2013) used fMRI to investigate TMS-evoked cortical activity in the motor areas. They delivered suprathreshold TMS to the left M1 or stimulated the right median nerve, and compared the fMRI responses. Sensory components only explained a small part of the TMS-induced activity in M1, indicating that fMRI combined with TMS to M1 can be used for functional imaging of motor networks.

Falciati et al. (2013) investigated whether motor evoked potentials (MEP) reflecting upper-limb cortical excitability were modulated during visually-guided saccades. They clearly showed that fast saccades toward a visual target were accompanied by changes in MEP amplitude. The results are in line with the viewpoint that gaze and limb control partially share a common neural system.

Two papers offer numerical models to explain changes in neural dynamics in response to brain stimulation. Chang et al. (2012) proposed a multivariate autoregressive model that describes interactions between cortical activities during direct electrical stimulation of the cortex, which was performed using implanted electrodes in patients with intractable epilepsy. The model-predicted responses matched well with real intracranial recordings. They also succeeded in assessing changes in the level of consciousness, estimating information integration in wakefulness and deep sleep using the model. Sato (2013) showed that the transient response of EEG theta-band activity to a theta-band photic flicker stimulation during memory encoding predicted the subsequent performance of memory recall. He proposed a numerical model in which this phenomenon is explained by the time constant of a driven harmonic oscillator that is smaller during successful encoding than during unsuccessful encoding.

Several articles have presented works that involve tDCS or tACS. Neuling et al. (2013) paper demonstrated state-dependent long-lasting aftereffects of tACS. They observed enhanced individual EEG alpha power for at least 30 min after tACS under eyes-open, low endogenous alpha-power conditions, whereas alpha power could not be further enhanced with tACS under eyes-closed, high endogenous alpha-power conditions. Marangolo et al. (2013) demonstrated that anodal tDCS over the left inferior frontal cortex (Broca's area) combined with “conversational therapy” improved speech production in patients with chronic aphasia. Fiori et al. (2013) investigated segregated tDCS effects on noun and verb naming and found that noun naming was improved after anodal tDCS over the temporal region, whereas verb naming was improved after anodal tDCS over the frontal region in aphasics. Both of these works show that it is possible to induce changes in altered brain dynamics, possibly leading to clinical recovery. Lapenta et al. (2013) demonstrated that tDCS over the left M1 modulated focal brain oscillations associated with motor imagery and movement observation. More specifically, they found that anodal tDCS over M1 led to mu-rhythm synchronization, whereas cathodal tDCS resulted in mu desynchronization.

There are four review papers on this topic. Parks (2013) summarized the current methodology for combining TMS with non-invasive near-infrared optical imaging techniques, such as functional NIRS and the event-related optical signal (EROS). Herrmann et al. (2013) reviewed tACS works mainly on oscillatory neural dynamics, physiological mechanisms, and modulation of brain functions, such as motor, perception, and higher cognitive processes. Saiote et al. (2013) reviewed studies that combined tDCS or tRNS with fMRI. They summarized and discussed results and the great potential of these methods to modulate human brain activity in a specific way. Carson and Kennedy (2013) contributed with a review paper on paired associative stimulation (PAS), focusing on prototypical forms of PAS in which single-pulse TMS is combined with peripheral nerve stimulation. They reviewed a lot of empirical evidence and interpretations of PAS effects in relation to spike-timing dependent plasticity mechanisms and concluded that additional explanatory models are required to go beyond the spike-timing dependent plasticity account.

There have been technical difficulties in using NIBS techniques together with imaging methods, and there is still a long way to go in the field before approaches such as online tACS-EEG recording become established. However, by virtue of recent developments in technical instrumentation and analysis, as can be seen in the TMS-EEG field, concurrent recordings have become not only possible but also very appealing. This Research Topic shows how we can now measure and analyze brain activity with these combined methods to probe the neural dynamics, brain state, excitability, plasticity, networking, and information flow in the intact human brain. Moreover, these combined methods can potentially show causal roles of neural dynamics in various brain functions. Taken together, manipulative and perturbational approaches with NIBS have great potential to give a better understanding of neural dynamics and functions of the human brain. We believe that the excellent contributions collected in this e-Book enable the reader to obtain new insights into non-invasive manipulation of human brain dynamics and will provide inspiration for future studies in this field of human neuroscience.

## Conflict of interest statement

Risto J. Ilmoniemi is founder, former CEO, scientific advisor, and minority shareholder of Nexstim plc. The other authors declare that the research was conducted in the absence of any commercial or financial relationships that could be construed as a potential conflict of interest.
